# “What matters to you?” A longitudinal qualitative study of Norwegian patients’ perspectives on their pathways with colorectal cancer

**DOI:** 10.1080/17482631.2018.1548240

**Published:** 2018-12-12

**Authors:** Frank Hansen, Gro K. Rosvold Berntsen, Anita Salamonsen

**Affiliations:** a National Research Center in Complementary and Alternative Medicine, Department of Community Medicine, Faculty of Health Sciences, UiT The Arctic University of Norway, Tromsø, Norway; b Primary care research group, Department of Community Medicine, Faculty of Health Sciences, UiT The Arctic University of Norway, Tromsø, Norway; c Norwegian Center for e-Health Research, University Hospital of Northern Norway, Tromsø, Norway; d RKBU North - Regional Centre for Child and Youth Mental Health and Child Welfare, Faculty of Health Sciences, UiT the Arctic University of Norway, Tromsø, Norway

**Keywords:** Colorectal cancer, longitudinal study, patient-centredness, person-centred care, supportive cancer care, illness trajectories, patient pathways

## Abstract

**Purpose**: Person-centred care (PCC) is a well-acknowledged goal throughout the western world both within the health care services sector and for the patients themselves. To be able to create a future health care system that includes improved PCC, we need more in-depth knowledge of what matters to patients, how “what matters” might change over time, and tentative descriptions of commonalities across patients’ perspectives. The aim of this study is to contribute to this knowledge base. **Methods**: We conducted a qualitative interview series over one year with nine Norwegian patients who were recently diagnosed with rectal cancer tumor-node-metastasis stage I–III. **Results**: We found that: (1) patients have an initial focus on “biological goals” and conventional treatment; (2) pathways are unique and dynamic; (3) family and friends affected patient pathways positively with respect to meaningfulness and quality of life, but for some participants also negatively because there were heavy burdens of caretaking; (4) receiving help in the health care system depended on the patients’ navigation skills; (5) pluralism in health-seeking behaviour was important in all patient pathways. **Conclusion**: Long lasting illness may be a dynamic and complex journey. These results represent some features of a pathway with cancer and are important because they contribute with knowledge about what matters most seen from the cancer patients’ point of view.

## Introduction

Studies have revealed that person-centred care (PCC) leads to better health (Coulter & Ellins, 2007; Coulter et al., 2015). It is therefore not only an ethical standpoint, but also pragmatically oriented towards best outcomes. This has led to PCC being called for by health authorities, ethicists, legislators, and patients alike (Helse og omsorgsdepartementet, 2001; Pellegrino & Thomasma, 1987; Taylor, 1992). The practice of person-centred treatment is here defined as acting according to patients’ values, preferences, and personal needs (Mead & Bower, 2000). Health care professionals should relate not only to medical knowledge, but also to people’s individual understandings of what is important to them as patients. Despite the importance placed on PCC, there are no qualitative longitudinal studies that have explored what matter most to patients who are in a long-term illness, and how this may change over the course of long-term diseases progression. This article is a part of the PATH study (Patients’ Accounts of Trajectories to Healing) with the overall aim to get insight into long-term illness from a patient perspective. Demographics, cancer stage and biomedical treatment of the participants are presented in Table 1.

**Table I.** Demographics, cancer stage and biomedical treatment of the participants in the PATH study.

Characteristics	Number of participants (n = 10)
**Gender**	
Female	5
Male	5
**Age**	
Unknown	1
25–44 years	0
45–65 years	6
> 65 years	3
**Education**	
Secondary education	3
High school or equivalent	2
Trade/Vocational diploma	3
Bachelor degree	0
Master’s/Professional degree	2
**Marital status**	
Married or living with a partner	7
Not married	3
**Living**	
Alone	3
With children	
With spouse/partner	5
With spouse/partner and children	2
**Work**	
Unknown	1
Employed part time	1
Self-employed	2
Unemployed	0
Retired	1
Disability/income	3
**Colorectal cancer stage, TNM**	
Stage 1	3
Stage 2a-b	3
Stage 3a-c	4
**Treatment**	
Preoperative radiotherapy	5
Surgical treatment:	
*Biopsy only*	1
*Bowel resection and re-anastomosis*	7
*Bowel resection with permanent stomy*	2
Post-operative chemotherapy	7

*One of the participants withdrew from participation in the project after the baseline interview.

### PCC and criticism of health care practices

The quality of treatment and care of patients depends on both clinical knowledge and knowledge about the patient’s character and personal life. Along with conventional treatment, these are important aspects of patients’ pathways to healing. Despite this acknowledgement,

from the late twentieth century there have been increasing critiques of the dehumanising aspect of contemporary medical practice. Criticism has focused on the displacement of the person of the patient by technologies, the redefinition of the patient in biomedical terms, and the distress suffered by the patients navigating a highly impersonal, overpressured health care system. (Parsons & Hooker, 2010, p. 345)

One of the earliest critics was Peabody. In his article “The Care of the Patients” published in 1927, Peabody was approaching some of the same challenges that we are trying to solve today:

The most common criticism made at present by older practitioners is that young graduates have been taught a great deal about the mechanism of disease, but very little about the practice of medicine—or, to put it more bluntly, they are too “scientific” and do not know how to take care of patients. (Peabody, 2015, p. 877)

Thus, a barrier to achieving the aim of care that is more person-centred in the professional sector is when the doctors do not see the person behind the diagnosis. Stange (2009) highlighted this issue when he quoted Osler, writing “it is more important to know what sort of person has a disease than to know what sort of disease a person has” (p. 102). It is therefore clear that the ideology of providing PCC is old, but the challenge of embedding it in everyday clinical practice still persists (Naldemirci et al., 2017; Riding, Glendening, & Heaslip, 2017). For example, a study found that doctors who were courteous often still systematically avoided dealing with the personal topics that patients brought up, even when they could be relevant to diagnosis and treatment (Agledahl, Gulbrandsen, Førde, & Wifstad, 2011).

### The problem of a fragmented health care system

Norwegian health authorities acknowledge that the present uncoordinated health care service is a challenge. For example, there are few elements in the current system that incentivize holistic needs. Fragmented health care systems such as this are especially a burden for patients with long-term illnesses and complex needs, and in Norway the number of such patients is growing (Regjeringen.no, 2008–2009).

Depersonalization and despair are some of the unintended consequences of well-intentioned actions, Stange (2009) argued in his critique of fragmented, disintegrated health care systems. He saw a holistic approach as key:

Viewing health care as an evolving whole instead of only as fragmented parts can help us to feel hope where now there is cynicism. Personalization and relationship where now there is detachment and isolation. Professional and corporate shared responsibility where now there is narrow self-interest. High value health care where now there is waste and inequality. Maximizing the opportunities for health and healing, and abiding when healing and health are not possible. (p. 102)

The best possible pathways depend on a system that supports health and healing. To reach the goal of PCC, we need a system that combines the pieces of a multidimensional life with illness into a purposeful whole.

### Patient pathways

Norwegian health authorities understand patient pathways as being “a holistic, coherent description of one or several patients’ contacts with different parts of the health care system during a period with disease” (Helsedirektoratet.no, 2017). This definition of a patient pathway focuses on the series of evidence-based interventions *within* conventional health care services. While this may be a useful template for treating single diseases, it does not meet the holistic needs of patients, as the biomedically motivated pathways of clinical care are only a subplot of the pathways experienced by the patients. We therefore propose that in order to provide care that is truly person centred, health practitioners need to adjust their understanding of what constitutes patient pathways to include a wider set of dimensions beyond just health care related events; that is to say, they must include “life events” as well:

The concept of health events includes events involving the patient and a health care provider, experiences of symptoms and adverse events, and patient-initiated health events, such as dietary change and exercise. The concept of life events includes events that the patients themselves define as important in their life. Such life events may or may not be related to the cancer diagnosis and cancer treatment. (Salamonsen, Kiil, Kristoffersen, Stub, & Berntsen, 2016, p. 1592)

By including both health events and life events in the understanding of a pathway, one manages to grasp both the episodes of treatment and other important aspects of the life lived with illness from a patient’s perspective (Salamonsen et al., 2016). Compared to the clinically based understanding, this definition not only includes the patient’s perspective, but also gives a richer longitudinal picture over time of what patients are going through.

### Contribution

Though there is one quantitative, longitudinal study that has measured changes in the quality of life of patients with long illness pathways (Chambers et al., 2012), we are not aware of any qualitative longitudinal studies that have focused on determining what is important to such patients over time. By following patients over time and listening to their accounts, we can generate knowledge about what is important to them, which in turn can lead to care that is more person centred. The findings of this study may be transferrable to a larger group of patients, namely severe, long-term illnesses with relatively acute starting points.

## Aims and research questions

This article aims to uncover knowledge that will support the transformation of care services to become person centred. We aim to offer rich descriptions of the participants’ lives lived with colorectal cancer and what they themselves emphasize as important. The research questions analysed in this article are:

*What is important for persons diagnosed with colorectal cancer during their patient pathways?*

*And, based on these findings, which significant features do the patient pathways share?*

The exploration of these questions leads us into a discussion of the implications of current understanding of the patient pathway with regard to PCC.

## Sample

To be included in the study, patients had to fulfil the following criteria: they had to be between 18 and 70 years old, have been diagnosed with rectal cancer tumor-node-metastasis stage I–III (Dukes A–C) within the last 6 months, and have completed their primary surgical treatment. Our sample was a small sample of a diagnostically relatively homogenous group of patients, although the clinical cancer stages and prognoses varied across the group. Due to potential recall bias, it could have been interesting to meet the participants before primary surgical treatment as well. However, this was not our intention when designing the project because (1) we did not want to include the terminal patients. We therefore had to wait until after primary surgical treatment to know the patients’ prognosis; (2) our main intention was to learn more about how patients shape their own pathways, and not so much the acute phase; and (3) ethically we did not want to be an extra burden for newly diagnosed patients. Furthermore, their residence had to be a maximum of 500 km from the University Hospital of Northern Norway (UNN). All potential participants were identified based on UNN’s electronic patient records in the autumn of 2011. Patients who fulfilled the criteria for inclusion received an invitation letter (N = 20). All participants were informed that participation was voluntary and that they could withdraw from the study whenever they wanted. While 10 patients signed a written informed consent form, 1 withdrew from the project after the baseline interview, leaving 9 participants aged between 54 and 68 years old who participated in the study.

## Method

### Choice of data-collection methodology

This research is based on a longitudinal qualitative approach, a method that “can add depth and understanding to health care research, especially on topics such as chronic conditions, adherence and changing health policies” (Grossoehme & Lipstein, 2016, p. 1).

All the participants were Norwegians situated in northern Norway. Two of the authors and a research assistant conducted baseline and quarterly qualitative interviews in 2011–2012. The participants were followed over a period of 1 year every 3 months. Altogether, 46 interviews were conducted. Five of the nine the participants wrote diaries that were the starting point for the quarterly interviews. For the remaining four patients, we employed a semi-structured interview guide to gather information regarding their health and life events since we last talked. The baseline interviews were conducted face to face, while the rest of the interviews were mainly conducted over the phone. The interviews were between 45 and 150 mins long. As a part of the PATH study, five of the participants took part in a workshop where, among other things, they were asked to make a drawing of their patient pathway.

We chose colorectal cancer patients as the focus of our study as cancer is a diagnosis with a somewhat obvious starting point and that stretches over time. Furthermore, cancer patients’ needs are often complex and the patients frequently initiate treatments outside conventional health care (Horneber et al., 2012). Cancer patients therefore make up a suitable group for the study of variation in patient pathways over time in complex and long-term contexts. We have used pseudonyms instead of the real names to represent participants in this study to protect their anonymity and confidentiality. Because cancer is a sensitive topic, this was both an ethical and practical choice in this study.

## Analysis

All the interviews were transcribed verbatim by a professional transcriptionist. We worked in an interdisciplinary research team consisting of two anthropologists, a medical doctor, and a medical sociologist. The analysis was based on an inductive approach. We first read the interview transcripts closely. Thereafter, we coded them in NVivo 11 Pro, using nodes to group relevant content from the wide range of experiences and processes that the patients described. The coding was mainly based on what the participants expressed as important in the interviews, that is to say they used the word “important” in a sentence or we explicitly asked “What has been the most important during this recent period of time?” Other content was coded based on the researchers’ interpretation of the materials where the participants implicitly spoke about aspects they felt were important to them over the last few months. Thus, even though we were interested in the stories in terms of gaining a larger picture, our analysis was not a narrative analysis approach as such. Rather, by using NVivo as an organizing tool, we were able to undertake a qualitative content analysis focusing on “characteristics of language as communication with attention to the content or contextual meaning of the text” (Hsieh & Shannon, 2005, p. 1278). Finally, it should be noted that in addition to the process above, we also created mind maps and infographics in order to visualize the analytical ideas we had as they developed. This was experienced as useful both for creating a clearness of thought and as a starting point of interdisciplinary discussions in the research group.

## Results

Based on our interpretation of what was important to the participants in our study, we found five categories that the participants emphasized.

(1) ***Initial focus on biological goals and conventional treatment***

The participants saw a conventional medical doctor after recognizing symptoms, and receiving the cancer diagnosis was by most of them described as a “shock.” At this early stage in the participants’ pathways their most important focus was on biological goals. The concept of biological goals is taken from Berntsen et al.’s creation of health concepts and goal typology: “Health is absence of biological malfunction or disease (Goals 1–5). Diseases have a biological basis or aetiology for symptomatology and signs. The goal for care is to remove the cause of disease and relieve symptoms through biological manipulation” (Berntsen et al., 2015, p. 4). Specifically, the participants emphasized early treatment at the local hospital as important, and that their goal was to become rid of the tumour at the point of diagnosis.

They furthermore all had trust in biomedical approaches to treating the cancer, and thus the participants chose conventional treatment and followed their physician’s advice. “I wanted to survive,” Jacob said when we asked him for his motivation for accepting conventional treatment. Elias had a similar answer: “The goal of the treatment is to live as long as possible and enjoy the time I have left,” he said. “I have stopped working. Now I will travel around and get lost.” We interpreted the choice of conventional treatment and listening to their doctor’s expertise as important for the participants at the moment of getting a potentially life-threatening diagnosis. For some, the medical treatment was explicitly underlined as the most important thing they did during their pathway.

Julie was a participant who also made use of herbal medicine from India and a special diet programme to cope with the cancer itself. She was motivated to follow this as her sole form of treatment, but returned to the conventional system when her doctors refused to postpone her operation. Based on our findings, we thus posit that a patient chooses the intervention that is culturally understood as the best thing to do according to one’s goals and the opportunities one has available. “I had no choice,” Per said, which can be interpreted as an understanding that there is only one way to survive cancer and that is by receiving conventional treatment.

After the initial conventional treatment, the participants struggled with experienced side effects or adverse events to various degrees. Permanent or temporary stoma, radiation injuries, fatigue, pain, and diarrhoea are some examples. “You pay a very high price to get well,” Julie said, who suffered from late injuries and side effects in terms of pain and fatigue. Those who only underwent surgical treatment reported fewer side-effects/adverse events than those who also received cytotoxic drugs and/or radiation therapy.

(2) ***Pathways are unique and dynamic***

The next category that emerged was that every pathway or “journey” was unique and dynamic. This is not surprising, as each person is different. They are unique genetically, have a unique repertoire of life experiences, and the cancer was experienced within their unique personal contexts.

Empirically we focused on the individual health and life events deemed important by the participants. In addition to having to cope with the cancer itself and the biological goals related to the disease, managing unforeseen life events not related to the disease was found to be a dominant issue for the participants. These include life course disruptions that may have happened *before* the cancer diagnosis and that had a significant impact *during* the participant’s pathway (Salamonsen et al., 2016). Sometimes life events were so dramatic that they overshadowed the concern about the cancer itself and the biological problems the participants experienced in their own bodies.

We uncovered such tendencies within the participants’ stories when we asked them the open-ended question regarding what had been most important to them over the last period of time. We have selected a few examples of such events. The examples are meant to illustrate how different pathways have different characteristics and must be understood individually.

The first example is how the loss of her daughter before diagnosis was *the* major topic in our conversations with Eva. The loss of a family member was also a major topic for Mari, whose husband had, some years ago, been diagnosed with the same type of cancer as herself. This was a man that she had taken care of for a long time because of the disease. When she received the diagnosis, she therefore already knew what was awaiting her. Furthermore, when she started her treatment and needed care most, her husband died. Later, she also suffered the loss of a child, and had complications with her treatment.

Another example is Per, who in the first post-surgical period of his pathway regretted having undertaken the operation, despite the fact that the operation had successfully excised the cancer. Prior to the operation, he knew he would have to have a stoma post-surgery, but had been assured by everyone that a stoma would not lead to any problems. That was not the reality for Per, however, as he not only faced unforeseen challenges with the stoma, but also experienced difficulties finding qualified help. Only by taking control and dealing with the health care system in an autonomous way, and by participating in self-management courses and learning through his own experiences, was he able to educate himself and become a “stoma expert.” He later began to share his experiences and knowledge with new stoma users in various discussion forums. “I tell new patients that a stoma may cause a lot of problems, because it can,” he said. In the last interview, he told us: “Now I can live with it.” The stoma was not a problem anymore.

Emma’s pathway was again different from the others’, as she was not concerned with her cancer at all: “After the doctor said I would be fine, I have not worried about it at all.” She told us her main concern, rather, was a fear of Alzheimer’s disease as it ran in her family: “I would rather die of cancer than fade away due to Alzheimer’s,” she said. She was also very concerned with the future of her son, who struggled with mental health issues. He moved in and out of her house during her pathway while he was trying to find a job.

Julie was diagnosed with secondary radiation injuries. She felt that if she had been able to try using herbs and the diet programme as treatment just a little longer before starting her radiation treatment, then she could have minimized the tumour and would have been spared the affliction of the radiation injuries. Suffering from intense pain, the most important thing for Julie during the first period of time was actually managing to sit and stand. Later she emphasized that the most important things for her were being at her cabin, being in nature, picking berries, and visiting her family. Early in the pathway she had a hope of getting back to work, but this later proved out of reach. “The doctor told me that I probably cannot go back to work,” Julie said. “The only thing I can do is reconcile myself to the situation as it is.” She now receives disability benefits from the government.

In contrast, other participants had very few complaints: Ken commented on the process, stating everything was going smoothly for him. In a similar manner, David told us that he had few complications after the treatments. All he really had to deal with were some problems with diarrhoea, which impacted some of his activities, such as going fishing.

Jacob was in general happy with the services he got from the hospital. He added, however, that

the health workers should always be reminded to see the whole person behind the diagnosis. Because even though the clinical pathways may be pretty similar regarding the prognosis and things like that, each case is unique and individuals are very different. To really see each person behind the diagnosis is important both for safety and coping for every patient. That again will have an impact on how good of an effect one actually gets from the treatment and how one copes with the pathway as a whole. When a person gets a potentially life threatening disease, the initial thought is death. The aspect of time is therefore very important both on an individual level, but also on a system level.

Jacob here comes to the centre of this second finding. Namely, that though there are generally two kinds of pathways as can be seen by the examples above, i.e., those that are relatively simple and those that are complicated, each patient pathway is unique. The implication for practitioners is to see the person behind the diagnosis

Entangled with the notion of unique pathways is the understanding that what is important to patients is dynamic and changes over time. Patients not only emphasize different things as being important, but each theme unfolds and changes with time. Specifically, we found that patients’ needs, preferences, and emotional statuses change individually over time during their pathway, and that the pathway encapsulates events related to both health and life.

3) ***Impact of family and friends in a pathway***

Family was of great importance for the participants, both existentially and pragmatically. Existentially, when you get a potentially life-threatening diagnosis such as cancer, thoughts may be led towards the end of life, and the very real possibility that you may not have much time left. This seems to provoke a realization of “what really matters” in life. Because of this, the participants emphasized that they became more conscious of cultivating their closest relationships during their pathway. For example, for Jacob, the thought of losing his life was equivalent to that of losing his family. This made him realize he needed to try to spend as much time with them as he could. Mari is another example of this, who stated that “material objects have no importance. What really matters is friends and family.” This “new” realization made her move from her home town to the place where her children and grandchildren lived.

It was not only becoming aware of their existential nature that occupied the participants’ minds as related to the importance of those they had close relationships with. They also emphasized these relationships as being an important part of their healing process. Jacob said, metaphorically, that he “went into a dark place” when he received the diagnosis. “It was a state of darkest darkness,” he told us. However, there was no psychiatrist involved in this depressive episode. “It lasted only a day or so,” Jacob said. “Family and friends helped me out of it.” For Ken, this practical and emotional support came not only from friends and family, but from colleagues as well. “They visited me at the hospital and even offered me economical support if it was needed.” Ken said that he was very moved by all the support and care that he received from the people around him.

The participants received practical help from their family and/or friends, and they told us that they received emotional support. However, there were also challenges linked to interactions between themselves and their family and friends. Per, for example, stated: “I don’t need anyone. I don’t need anyone to feel sorry for me. It will only make the condition worse.” Instead of keeping family close, he preferred to keep a distance. He preferred to stay alone. He did, however, attend a self-management support centre for cancer survivors, where he found pleasure in the relationships he built. “I felt better when I heard that others were also suffering. It was not only me,” he laughed. For Jacob it was difficult to communicate the diagnosis to his children, and this could had been done differently, he said. Another informant worried about how the children would be treated at school when they had a father with stoma and colorectal cancer, conditions associated with stigma.

Overall, we found the role of close relationships was highlighted as being very important in a cancer pathway and this important role manifested itself in various ways, giving both meaning and healing in the context of cancer. “Care and love are important words during an illness pathway,” Mari said:

It is a unique experience when a child says “Grandmother, wake up! I love you!” All the love and good things the heart can be filled with. You are privileged to be there present with your grandchild, to have walks and explore the world together with her, to see a little flower, a mosquito, and a snail.

4) ***Navigation in the national health care system***

We define “navigation” in the national health care system as a type of knowledge or skill that enables patients to find qualified help. To receive proper help when needed is crucial for patients’ health and healing. Failure in care navigation creates extra burdens for the patients by making them frustrated and feel lost, and waiting for treatment is associated with anxiety for both patients and their peers.

The participants praised the hospital’s cancer treatment as swift, and they felt they had been highly prioritized. Several of these worked in the health care system themselves, and thus used their professional network to get information and to get things done. Take Mari, who was a health care worker, as an example: she made use of her professional network to get the best surgeons. Coming from within the system also meant she could benefit from her existing knowledge regarding how the national health care system works. She basically had a map and compass with which to navigate the “jungle” of the system. However, despite being content with receiving quick treatment and help, participants felt the processes with regard to receiving test results were too slow, causing them to feel worry and stress.

Participants had to “fight” to get the help they needed, and some often felt lost in the system if they did not know the system from within. Elias, for example, chose to go outside the national health care system and make use of a private doctor to get an earlier examination date. “It would have been six months waiting if I had gone through my family doctor, while it was only two weeks at the private office,” he said. This made him feel it was an unfair system. Later in his pathway, however, Elias had problems getting help in conventional health care. Looking back, we asked him what had been most important during the pathway. “When you get the diagnosis, things are spinning around in your head. You think about this and that, and then you don’t know where to enquire to get things done.” What Elias felt impacted him the most was the lack of knowledge regarding how to navigate the system to get proper help.

Per is another example of a patient who struggled to get proper help. He had to use creativity and “backdoors” to get the care he needed when he was suffering from problems related to his stoma, which created extra stress and burdens for him during a time that was already very difficult. Similarly, Emma experienced the health care system as being slow (long waiting times), complicated, and difficult to navigate as related to her post-operative hernia.

A topic related to navigation is how the national health care services communicate with their patients. Emma thought that the letters from the hospital were cryptic, with Latin words, etc. “I went to my doctor to get the letter translated,” she said, “but even he did not understand everything.” She also got an erroneous letter at one time with information about a tumour in the brain. It was a disturbing experience. Letters from the hospital often led to misunderstandings and worries for the receiver.

“One-time-doctors,” as Elias called them, were another concern with regard to the system. “You have to tell your story over and over again,” he said. “It is a new doctor every time.” Not only was it a burden to repeat one’s story, but Elias experienced that doctors also met him unprepared with regard to his history. This complicated the consultations and was experienced as frustrating.

5) ***Pluralism in health-seeking behaviour***

The participants used a combination of approaches on their path to health and healing. Elias said at one point that “I will not try any witchcraft.” Here “witchcraft” (*heksekunster* in Norwegian) should be interpreted as any kind of practice based on superstitions. When we asked him if he had tried any treatment outside bio-medicine he answered “No.” But continued: “Maybe I have called someone,” and laughed, “someone who you could say has some special abilities.” He confirmed that he had both called and met face to face with two traditional healers or “readers,” which is the direct translation of the Norwegian term *leser*. The practice of “reading” was not considered witchcraft by Elias, as readers, in his cultural logic, belong to a category of professional individuals that hold healing knowledge and power, where it is understood that God acts through the reader. “It does not hurt to try,” he said.

“I will try everything to get well again.” Julie’s attitude towards getting well can be categorized as epistemological individualism. She explored each possibility she came across and learned through experience. We already mentioned above that she tried to deal with the tumour using herbal medicine and a special diet. She also made use of healing and acupuncture outside the conventional health care system.

Mari received healing from two different healers, and underwent a chakra balancing. She had conversations with both the hospital priest and psychiatrist. “They complement each other,” Mari said. They represented existential and psychological aspects of the respective experiences she had gone through. Various objects, such as an angel, a cross, and a praying cloth, were important to Mari. “I hold them in my hand when I need strength,” she said. “They are gifts from people who wish me all the best.” Mari also found it useful to take frequent walks, especially in nature: “Nature gives me inner strength.” She further showed us how she applied positive thinking to negative situations. “I looked at the cytotoxic drug. I thought of it as golden drops,” she said. “Golden drops that are going to heal me.” Psycho-motoric physiotherapy was another treatment that Mari emphasized as important in her pathway to healing. “We work with the physical as well as balancing the thoughts,” she told us.

Per, David, Ken, and Jacob did not use any alternative treatments, according to their understanding of the concept. However, they undertook a combination of approaches to intentionally improve their health after the diagnosis. For instance, diet change, courses to generate knowledge, and various physical activities. We would place these types of activities in the category of “self care.” The point here is to show that there is a complexity of actions on the way to better health in the context of cancer, and that these activities belong both inside and outside the realm of the conventional health care system.

## Discussion

We started the introduction by saying that PCC is a goal for patients, health authorities, legislators, and professionals alike, and argued that the development of PCC and better pathways should be based on medical understanding as well as the patients’ perspective of what is important. This is in line with Donabedian (1988) who suggested that assessing the quality of health care involves a three-part approach that consists of evaluating the health care’s structure, processes, and outcomes. This assessment approach is necessary because a good structure increases the likelihood of good processes, and good processes increase the likelihood of good outcomes. Thus, by considering all three factors a more complete assessment can be made (Donabedian, 1988).

In the above approach, processes include “the patient’s activities in seeking care and carrying it out as well as the practitioner’s activities in making a diagnosis and recommending or implementing treatment” (Donabedian, 1988, p. 1745). They are thus the sum of all the health-seeking actions in a pathway. Donabedian further made a distinction between technical processes, which are related to the biomedical diagnosis and treatment, and interpersonal processes, which refer to the relational quality of the care delivered, and highlighted how interpersonal processes impact the quality of the technical processes. That is to say, it is through interpersonal exchanges that the patient communicates information that the medical doctor needs to arrive at the correct diagnosis and “preferences necessary for selecting the most appropriate methods of care” (Donabedian, 1988, p. 1744). Furthermore, “privacy, confidentiality, informed choice, concern, empathy, honesty, tact, sensitivity—all these and more are virtues that the interpersonal relationship is expected to have” (Donabedian, 1988, p. 1744). Despite the importance of managing the interpersonal process, however, it is often ignored in the evaluation of health care. Perhaps part of the reason it is ignored is that it is quite challenging to implement PCC in clinical practice (Riding et al., 2017). One way of moving towards the goal of PCC may thus be to start by increasing the focus and understanding of the concept of the patient pathway, since the way we understand the world and act in the world are based on our concepts (Lakoff & Johnson, 1980).

### PCC and the patient pathway concept

The common medical understanding of a pathway as a series of clinical interventions and standardized packages for diagnosis gives a limited picture of relevant information if our aim is to provide patients with care that is more person centred. We referred above to Norwegian authorities’ definition of a patient pathway as “a holistic, coherent description of one or several patients’ contacts with different parts of the health care system during a period with disease” (Helsedirektoratet.no, 2017). However, our findings show that contact with the health care system is only one of the factors patients emphasize when discussing their pathways. This thus begs the question of whether the above definition provides a sufficient understanding of a patient pathway.

Studies have revealed that PCC leads to better health (Berghout, van Exel, Leensvaart, & Cramm, 2015; Coulter & Ellins, 2007; Matalon, 2008; World Health Organization, 2015). PCC, here defined as acting according to patients’ values, preferences, and needs (Mead & Bower, 2000), should also be practised because it is ethically correct (Pellegrino & Thomasma, 1987). Patients with chronic diagnoses have pathways stretching over long periods of time, where health care workers acting according to the patients’ values, preferences, and needs may be a significant factor in improving the patients’ well-being and causing them to experience their care as high quality. This leads to the conclusion that an understanding of the patient pathway as being a purely clinical journey does not fit within the concept of PCC. We thus suggest that the concept of a person-centred pathway may be useful when describing the journey a patient with a long-lasting disease embarks on after diagnosis. By introducing this concept, we move towards an understanding of a pathway that consists of both health and life events (Salamonsen et al., 2016). Not only will the route within conventional health care be present, but so will other matters that influence the patients’ condition. Such a concept allows for an implementation of a more holistic understanding that supports PCC, where it contains the perspectives of the professionals as well as the patients.

Stange (2009) reminded us about the problems of a fragmented health care system, highlighting for us how the benefits of finding new ways of thinking and acting about patient care are numerous. However, Stange did not include the aspect of time, which is a key factor when considering pathways. That is to say, a pathway is characterized by many dimensions that are in flux and flow over time. This is seen in the results of our study, where all of the participants used a combination of approaches to cope with their ever-changing situation. There is thus a need to implement care that takes into account the impermanent realities of patients. This will require a dynamic model rather than a static understanding, because a pathway is not only a puzzle with pieces creating a picture, but rather closer to a film evolving and changing over time.

## Methodological considerations

### Quality of data gathered through qualitative interviews over time

When employing qualitative interviews, as we have done in this study, it is important to be aware that the researcher’s presence may affect the data he or she is gathering. For example, respondents may undertake impression management, controlling what kind of information they give the researcher and what they choose to keep “backstage,” hidden for the “audience” (Berreman, 1962). This is different from long-time ethnographic fieldwork where participant observation is the most common approach, such as is commonly used in anthropology “to grasp the native’s point of view, his relation to life, to realize his vision of his world” (Malinowski, 1922, p. 25). With this in mind, it is interesting to compare the ethnographic method, which this study’s first author is familiar with through his background and experience as an anthropologist, with the qualitative interviews in this study.

The material is characterized by people who are open and sincere in sharing their story. We believe that a reason for this is that we held the role of interviewers and not therapists or peers. Thus many of the participants viewed us as neutral persons, which may be why they felt comfortable and safe sharing backstage information with us. Some explicitly told us that they shared aspects of their lives that they had not told anyone before. “I don’t throw pearls to swines,” Mari said, and emphasized that she was very careful with whom she spoke about complementary and alternative medicine and spiritual matters. Despite the fact that many are met with condemnation when they speak about these topics, they opened up to the interviewers. Some of the participants further used the interviewers to vent their worries and concerns, and some felt relief caused by telling their stories and reflecting over the questions. Such access to the inner life of the participants gives credibility to our data. We can thus conclude that the in-depth and open-ended interviews made us reach our goal, namely to come close to life lived and experience in the context of cancer diagnosis.

Another important factor that helped build trust between the interviewers and participants was that it was a longitudinal study where we followed the participants over a whole year, as was suitable for a pathway study. The trust built over time also affects the depth and transparency of information the participants are willing to share, i.e., it minimizes the level of impression management. Furthermore, by clearly communicating our aim of the study—to learn more about cancer patients’ pathways in order to improve future health care—we were able to strengthen the degree of openness and completeness of the responses, as all the participants hoped to help us with this goal. The participants contributed with their perspectives as they wished to help improve future health care and the services provided to future patients.

### Validity of sample

Our sample was a small sample of a diagnostically homogenous group of patients. The material was balanced in terms of gender, but all of the informants were adults from northern parts of Norway. Ideally, it would have been interesting to have a more widespread sample with regard to variation in age and cultural background. Another approach could have been to strategically choose patients with particular experiences to go deeper into certain phenomena. For instance, the use of complementary and alternative medicine in a long-term pathway. Despite these limitations, we show that, even though the diagnosis is the same, the individual pathways are unique, while at the same time they share certain patterns or common features.

### Validity of coding

The findings are based on codings in NVivo of the participants’ own accounts of what has been important and meaningful during their pathway with cancer. The strategy of categorizing sentences and questions with the word “important“ and the summative approach of content analysis was aimed at bringing credibility to the study. This approach also required interpretations, which may always be subject to misinterpretation. The co-authors thus reflected upon the first author’s coding to ensure a common understanding of coding-concepts across authors. In the writing process, we included an extensive use of quotes to exemplify the participants’ perspectives.

### Reflexivity

The research group consisted of two anthropologists, a sociologist, and a medical doctor. This composition of interdisciplinary researchers provided a beneficial cooperation that generated knowledge about long-term pathways from a patient perspective. A potential drawback of our team make-up, however, was that our cultural backgrounds were all the same as the participants’. The challenge with studies of our own societies is that it may make the researcher “blind” as there are many cultural ideas and practices taken for granted. We believe this was minimized in the current study as looking into detailed descriptions of life lived with cancer is like studying a different world. It should additionally be noted that deep knowledge about the society in question was at the same time an important resource for the project. All the authors read the interview material. We discussed NVivo coding, synopsis of the stories, and illustrations. An ongoing reflection led to the findings in this article. This process would have been much more difficult and less effective without this common deep knowledge.

A final point regarding the make-up of the research group is that the first author had no previous cancer-related experience or insight, while the others had worked with health and illness issues and aspects surrounding cancer before. Overall, the different backgrounds of the researchers in the group resulted in dynamic and interesting discussions.

## Conclusion

The results of this study are important because they contribute knowledge about what matters to patients during their pathway with a serious illness such as cancer. First, the results show that life with cancer affects different aspect of human beings and their relationships. Challenges related to health and illness need to be met with a combination of approaches, both inside and outside the conventional health care system. Second, each pathway is dynamic and evolves in a different way. We therefore argue that a pathway is characterized by its impermanent qualities as it moves through time and space.

PCC is about seeing the person and not only their diagnosis. It is about understanding the individual experience of illness in their unique life context. As we have shown, there are both ethical and pragmatic reasons to work towards implementation of this in the health care system. The aim of achieving PCC therefore leads us in this pathway study to suggest the need for a new pathway concept. This is because the previous understanding of a pathway as a series of contacts with the public health care system is incomplete as it not only does not comprise patients’ perspectives, but it also does not include aspects related to patients’ life events or the combination of approaches patients undertake with intention to heal or improve their various health issues. We thus suggest that a way to facilitate change in future health care services so that it includes improved PCC in practice may be to start with the language. We suggest that the concept of a “person-centred pathway” may contribute to this aim. This concept includes both health events and life events that are important to the patients themselves. A person-centred pathway will therefore respect patients’ values, preferences, and needs. Furthermore, this understanding must also imply a flexible approach by the social health care system, as person’s values, needs, and preferences may change during a pathway.

Overall, a person-centred pathway is a pathway where the patient’s individuality in terms of context, life-experience, and life-beliefs are all taken into account. It is a process where patients are seen and heard, and where “what is important to them” is always at centre stage in the process. Applying a person-centred pathway has the potential to humanize and defragment the care experience.
